# Retinal ferroptosis as a critical mechanism for the induction of retinochoroiditis during ocular toxoplasmosis

**DOI:** 10.1016/j.redox.2023.102890

**Published:** 2023-09-17

**Authors:** Kazuhisa Yamada, Akira Tazaki, Nanako Ushio-Watanabe, Yoshihiko Usui, Atsunobu Takeda, Masaaki Matsunaga, Ayana Suzumura, Hideyuki Shimizu, Hao Zheng, Nanang R. Ariefta, Masahiro Yamamoto, Hideaki Hara, Hiroshi Goto, Koh-Hei Sonoda, Koji M. Nishiguchi, Masashi Kato, Yoshifumi Nishikawa, Shinya Toyokuni, Hiroki Kaneko

**Affiliations:** a Department of Ophthalmology, Nagoya University Graduate School of Medicine, Nagoya, 466-8550, Japan; b Department of Occupational and Environmental Health, Nagoya University Graduate School of Medicine, Nagoya, 466-8550, Japan; c National Research Center for Protozoan Diseases, Obihiro University of Agriculture and Veterinary Medicine, Obihiro, 080-8555, Japan; d Department of Ophthalmology, Tokyo Medical University, Tokyo, 160-8402, Japan; e Department of Ophthalmology, Graduate School of Medical Sciences, Kyushu University, Fukuoka, Japan; f Department of Public Health, Fujita Health University School of Medicine, Toyoake, 470-1192, Japan; g Department of Pathology and Biological Responses, Nagoya University Graduate School of Medicine, Nagoya, 466-8550, Japan; h Department of Immunoparasitology, Research Institute for Microbial Diseases, Osaka University, Suita, Osaka, Japan; i Department of Biofunctional Evaluation, Molecular Pharmacology, Gifu Pharmaceutical University, Gifu, 501-1196, Japan; j Center for Low-Temperature Plasma Sciences, Nagoya University, Furo-Cho, Chikusa-ku, Nagoya, 464-8603, Japan

**Keywords:** Toxoplasma, Ocular toxoplasmosis, *T. gondii*, Retinal ferroptosis, Iron, Toxoplasmic retinochoroiditis, Vitreous humor

## Abstract

Toxoplasmosis is a major infectious disease, affecting approximately one-third of the world's population; its main clinical manifestation, ocular toxoplasmosis (OT), is a severe sight-threatening disease. Nevertheless, the diagnosis of OT is based on clinical findings, which needs improvement, even with biochemical tests, such as polymerase chain reaction and antibody detections. Furthermore, the efficacy of OT-targeted treatment is limited; thus, additional measures for diagnosis and treatments are needed. Here, we for the first time report a significantly reduced iron concentration in the vitreous humor (VH) of human patients infected with OT. To obtain further insights into molecular mechanisms, we established a mouse model of *T. gondii infection*, in which intravitreally injected tracer ^57^Fe, was accumulated in the neurosensory retina. *T. gondii*-infected eyes showed increased lipid peroxidation, reduction of glutathione peroxidase-4 expression and mitochondrial deformity in the photoreceptor as cristae loss. These findings strongly suggest the involvement of ferroptotic process in the photoreceptor of OT. In addition, deferiprone, an FDA-approved iron chelator, reduced the iron uptake but also ameliorated toxoplasma-induced retinochoroiditis by reducing retinal inflammation. In conclusion, the iron levels in the VH could serve as diagnostic markers and iron chelators as potential treatments for OT.

## Highlights

• The iron concentration in the vitreous humor of OT patients was reduced.
• Retinal ferroptosis was observed in *T. gondii*-infected mouse eyes.
• Deferiprone prevented OT by reducing iron accumulation in the retina.

## Graphical abstract

**Figure:**
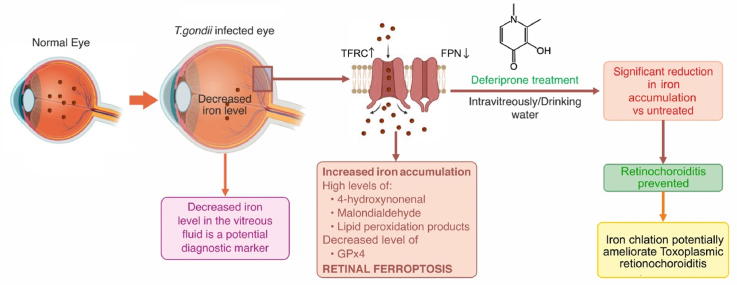

## Abbreviations

AIDS: Acquired immunodeficiency syndrome
ARN: Acute retinal necrosis
AH: Aqueous humor
DMT: Divalent metal transporter
DW: Drinking water
ELISA: Enzyme-linked immunosorbent assay
Fpn: Ferroportin
Fth: Ferritin heavy chain
Ftl: Ferritin light chain
GPx4: Glutathione peroxidase 4
HSV: Herpes simplex virus
IL6: Interleukin 6
INL: Inner nuclear layer
IS: Inner segment
i.v.: Intravenous injection
IVT: Intravitreal injection
LA–ICP–MS: Laser ablation inductively coupled plasma mass spectrometry
MDA: Malondialdehyde
MH: Macular hole
ONL: Outer nuclear layer
OS: Outer segment
OT: Ocular toxoplasmosis
PDR: Proliferative diabetic retinopathy
PFA: Paraformaldehyde
POI: Post infection
PR: Photoreceptor
RGC: Retinal ganglion cell
ROS: Reactive oxygen species
PR-IS: Photoreceptor inner segment
PR-OS: Photoreceptor outer segment
RT-PCR: Real-time polymerase chain reaction
TEM: Transmission electron microscopy
TFRC: Transferrin receptor
VH: Vitreous humor
VZV: Varicella zoster virus
4-HNE: 4-Hydroxy-2-nonenal

## 1 Introduction

Toxoplasmosis, a zoonosis caused by infection with the intracellular parasitic protozoan *Toxoplasma gondii,* is estimated to affect approximately one-third of the world population [1]. Ocular toxoplasmosis (OT) is one of its major clinical manifestations. The prevalence of OT varies regionally, but in specific areas of South America, 17% of the population is reportedly affected [2]. Toxoplasmic retinochoroiditis, the main lesion of OT, is characterized by acute necrotizing retinochoroiditis and accounts for 28%–50% of all cases of posterior uveitis. Moreover, visual loss occurs in 27% of the patients with OT, leading to legal blindness in ≥1 eye in 24% of the patients with OT [3].

The diagnosis of OT is based mainly on the clinical observation of focal necrotizing retinochoroiditis. The combination of clinical findings and biochemical tests, e.g., antibody detection in aqueous humor (AH) or vitreous humor (VH), is generally sufficient to achieve a satisfactory diagnostic result [4]. Even in atypical cases or cases with uncertain diagnosis, antibody titers, including intraocular antibody production (Goldmann–Witmer coefficient) and polymerase chain reaction (PCR) of AH or VH, are useful with high specificity [5,6]. However, while being one of the most reliable diagnostic methods, the PCR test conducted with the AH sample reportedly has a detection rate of <30% [[7], [8], [9], [10], [11]]. Since differences in the level of medical care may affect the control of infectious diseases [12], it is desirable to improve the diagnosis rate without using highly specialized equipment. Previous studies have shown that *T. gondii* and other microorganisms require iron for replication and survival [13,14]. In contrast, the host possesses innate mechanisms to prevent microbial deprivation of their iron reserves because deprivation of the nutrient iron for microorganisms is one of the host's alternative methods to defense against bacterial and eukaryotic pathogens [[15], [16], [17]]. Iron is required in various important biological processes, including oxygen transport, DNA synthesis and adenosine triphosphate generation; thus, cells must contain enough iron. However, excess iron can generate reactive oxygen species, causing oxidative stress. Therefore, strict iron concentration control is critical both for cell survival and death [18,19]. Furthermore, ferroptosis or iron-related regulated necrosis [20] is reportedly caused by lipid peroxidation and occurs without the involvement of caspases, necrosome components, cyclophilin D, and the molecular machinery for autophagy [[20], [21], [22], [23]]. In the present study, we observed that the VH from human patients with OT showed significantly lower iron levels than other retinal diseases. We then established a mouse model of OT and revealed the involvement of the ferroptotic process. We further found that an iron chelator, deferiprone, improved OT in the mouse model.

## 2 Materials and methods

### 2.1 Sample collection and electrolyte measurements

For this study, we collected VH samples from the affected eyes of patients with macular hole (MH), a typical noninflammatory and noninfectious retinal disease, proliferative diabetic retinopathy (PDR), a noninfectious disease reportedly associated with chronic inflammation, acute retinal necrosis (ARN) caused by herpes simplex virus (HSV) or varicella zoster virus (VZV), and OT. We collected VH samples using a 25-gauge cutter and a RESIGHT surgical microscope (Zeiss, Oberkochen, Germany) by dry vitrectomy (non-irrigation) at the beginning of vitrectomy surgeries, which we immediately stored at −80 °C as previously described [24,25]. After centrifugation, we collected the supernatants and stored them at −80 °C until further use. We froze the VH samples and thawed them before measuring Fe with a LABOSPECT 008 (Hitachi High-Technologies Corporation, Tokyo, Japan), a calorimetric assay based on the 2-nitroso-5-[N-n-propyl-N-(3-sulfopropyl)amino]phenol (nitroso-PSAP) method.

This study was conducted in accordance with the guidelines of the Declaration of Helsinki; the protocol was registered within the UMIN Clinical Trial Registry (registered number UMIN000024553) and approved by the Nagoya University Hospital Ethics Review Board (2013-0010, 2022-0598). Written informed consent was obtained from all participating patients.

### 2.2 Human retinal sections

Formalin-fixed, paraffin-embedded human retinal sections from eyes with OT were obtained from the Doheny Eye Institute at the University of Southern California. Diagnoses were made based on histological evidence and clinical history. Sections were stained with hematoxylin and eosin or Berlin blue and a nuclear fast red dye. Normal control donor eyes were purchased from the Minnesota Lions Eye Bank (Minneapolis, MN, USA) and San Diego Eye Bank (San Diego, CA, USA), and formalin-fixed, paraffin-embedded sections were prepared.

### 2.3 Animals

All animal experiments were approved by the national research center for protozoan diseases, Obihiro University of Agriculture and Veterinary Medicine (permit number: 22-152), and performed in accordance with the guidelines of the Association for Research in Vision and Ophthalmology Statement for the Use of Animals in Ophthalmic and Vision Research. *T. gondii* (1 × 10^3^ tachyzoites of type II, PruΔku80Δhxgprt or type I, RH-GFP) [68] was intraperitoneally injected in 6–8-week-old C57BL/6J mice from Clea Japan (Tokyo, Japan). Cryopreserved mouse retinal sections were stained with FeRhoNox-1 (Goryo Chemical, Inc., Sapporo, Japan), specific for catalytic ferrous iron (Fe^2+^) [26] with 4',6-diamidino-2-phenylindole. The natural abundance ratios of ^56^Fe and ^57^Fe are ∼92% and ∼2%, respectively [27]. We injected ^57^Fe into the vitreous cavity (Intravitreal injection, IVT) or tail vein (intravenous injection, i.v.). For ^57^Fe IVTs, 1 nmol/1 μL of ^57^Fe (Processed into FeSO_4_, IRON-57 METAL Cambridge Isotope Laboratories, Inc. Andover, MA, USA) was injected using a 33-gauge needle (Ito Corporation, Shizuoka, Japan) as previously described [28] at day 0 soon after *T. gondii* (1 × 10^3^ tachyzoites of type II, PruΔku80Δhxgprt) [29,30] intraperitoneal infection. With respect to ^57^Fe i.v., 200 nmol/200 μL of ^57^Fe (processed into FeSO_4_) was injected at day 0 after *T. gondii* (1 × 10^3^ tachyzoites of type II, PruΔku80Δhxgprt) infection; the mice were euthanized at day 7 (7d POI model) or injected with additional ^57^Fe at days 0, 7, 14, and 21 and euthanized at day 28 (28d POI model). To determine the therapeutic effect of iron chelators against *T. gondii* infection in the eye, the mice were intravitreally injected (IVT) with 1.5 nmol/1 μL of deferiprone (DFP, Santa Cruz Biotechnology, Dallas, TX, USA) soon after *T. gondii* infection or treated per os (by mouth) with 1 mg/mL DFP in drinking water over 3 days before *T. gondii* infection and euthanized at day 7.

### 2.4 Laser ablation inductively coupled plasma mass spectrometry (LA–ICP–MS)

Endogenous ^56^Fe in the unstained human retina sections from patients with OT as well as ^56^Fe and ^57^Fe in *T. gondii*-infected or noninfected mice were detected by LA–ICP–MS as previously described [31,32]. In brief, the laser ablated the retinal section at a 10-μm-diameter spot on the surface. The counts of carbon (C), phosphorus (P), ^56^Fe, and ^57^Fe in each 10-μm square were quantified. P, ^56^Fe, and ^57^Fe were adjusted C counts to make a per-specimen correction for the amount of laser-evaporated tissue. Then, the images were constructed from P, ^56^Fe, and ^57^Fe values (C as reference value) obtained from each 10-μm square. The images were Gaussian blurred using the built-in software. For statistical comparison, retinal areas for the detection of ^56^Fe and ^57^Fe abundance were randomly selected from each retinal section.

### 2.5 Immunohistochemistry

Cryoprotected retinal sections (10-μm thick) were prepared and fixed in 4% paraformaldehyde (PFA), followed by incubation with rabbit antibodies against the transferrin receptor (Tfrc, ab214039; Abcam), ferritin light chain (Ftl, ab69090; Abcam), ferritin heavy chain (Fth, ab65080; Abcam), divalent metal transporter (Dmt1, ab55735; Abcam), and ferroportin (Fpn-1, NBP1-21502, Novus Biologicals, Littleton, CO, USA) and secondary antibodies labeled with Alexa Fluor 488 (Thermo Fisher Scientific) and DAPI (Invitrogen). The sections were analyzed using a fluorescence microscope (BZ-9000; Keyence Corporation of America). To detect and visualize ^57^Fe localization and its relation with cone photoreceptor cells in the retina via LA–ICP–MS, retinal sections of ^57^Fe IVT mice were fixed with 4% PFA and incubated with an anti-opsin antibody (AB5405, 1:200, Chemi-Con, Temecula, CA), followed by incubation with a colloidal gold-conjugated goat anti-rabbit polyclonal secondary antibody (1:20, Jackson ImmunoResearch Laboratories, West Grove, PA).

### 2.6 Transmission electron microscopy

Mouse retinal sections were fixed with 2% glutaraldehyde, followed by 2% osmium tetroxide for 3 h. Sections were dehydrated through an ethanol series and propylene oxide, and embedded in EPON 812 (TAAB Laboratories Equipment Ltd., Berkshire, UK.). Ultrathin sections (70–80 nm thick) were cut on an EMUC7i ultramicrotome (Leica Microsystems, Wetzlar, Germany), stained with 2% uranyl acetate and lead stain solution, and examined under a JEM-1400 Plus transmission electron microscope (Jeol Ltd., Tokyo, Japan).

### 2.7 Oxidative stress analysis

To assess the extent of lipid peroxidation, we examined the levels of 4-hydroxy-2-nonenal (4-HNE) and malondialdehyde (MDA). Immunostaining was performed with anti-4-HNE monoclonal antibodies HNEJ-1 and HNEJ-2 (2.5 μg/mL, mixed 9:1) [33,34].

MDA was quantified using an OxiSelec MDA Adduct Competitive ELISA Kit (Cell Biolabs, San Diego, CA) according to the manufacturers’ protocol. To assess antioxidant protein expression, mouse retinas were evaluated using anti-GPx4 antibody (#760-228, 1:80, Cayman Chemical, Ann Arbor, MI, USA) via immunohistochemistry.

### 2.8 Western blotting

For total protein collection, mouse retinas were lysed in RIPA buffer (Sigma-Aldrich) with a protease inhibitor cocktail (Roche Diagnostics, Indianapolis, IN, USA). We used the TaKaRa BCA Protein Assay Kit (Takara Bio Inc., Shiga, Japan) to determine protein concentrations. Proteins (10 μg) were run on SDS precast gels (Wako, Osaka, Japan) and transferred to PVDF membranes. These membranes were washed with PBS-T and blocked with 5% skim milk/PBS-T at room temperature for 2 h. The membranes were incubated with an anti-glutathione peroxidase 4 (GPx4) antibody (1:5000; ab215066; Abcam, Cambridge, UK) or β-actin (1:1000, Cell Signaling Technology, Massachusetts, USA) antibody at room temperature for 1 h. Then, an anti-rabbit HRP-linked secondary antibody was incubated (1:3000, Cell Signaling Technology) at room temperature for 1 h. The signal was visualized using enhanced chemiluminescence (ECL prime; GE Healthcare, Piscataway, NJ, USA) and captured using a ChemiDoc XRS + System (Bio-Rad, California, USA). The intensity of bands was quantitated *via* densitometry as previously described [35].

### 2.9 ELISA and real-time quantitative PCR

Retina lysates were prepared from *T. gondii*-infected mice with or without IVT of 1.5 nmol/1 μL DFP. Interleukin-6 (IL-6) protein level was measured using an ELISA kit (M6000B, R&D Systems) as previously described [36]. We used a value of “0” for samples under the detection sensitivity in the statistical analysis. The total RNA was reverse transcribed using the Transcriptor Universal cDNA Master Kit (Roche Diagnostics), starting with 2 μg of total RNA from each sample. RT-PCR was performed using the Thunderbird Probe qPCR Mix (Toyobo Life Science, Osaka, Japan) and gene expression assay containing primers and an FAM dye-labeled TaqMan probe for detecting mice *Tfrc*, *Fth*, *Ftl*, and *Gapdh* or a SYBR green qPCR kit and primers for *Fpn-1*, *Dmt-1*, and *Gapdh*. RT-PCR cycles using TaqMan consisted of a pre-denaturation step at 95 °C for 2 min, followed by 45 cycles of denaturing steps at 95 °C for 15 s, and annealing and extending steps at 60 °C for 60 s. RT-PCR cycles using SYBR green consisted of a pre-denaturation step at 98 °C for 2 min, followed by 45 cycles of denaturing steps at 98 °C for 10 s, annealing at 55 °C for 10 s, and extending steps at 68 °C for 30 s using QuantStudio 5 (Thermo Fisher Scientific). The relative expression of the target genes was determined using the 2^-△△Ct^ method. The TaqMan probes and primer sequences are listed in Table 1.

**Table 1:** Primer sequences used in this study.

Species	Gene Symbol	Gene name	Taqman Probe	Forward Sequence	Reverse Sequence
Mouse	Dmt	divalent metal transporter		5'-ggctttcttstgagcattgccta-3'	5'-attcgacgagacccacgagg-3'
	Fpn	ferroportin		5'-ttgcaggagtcattgctgcta-3'	5'-tagttaccacacgtcttgaggt-3'
	Tfrc	transferrin receptor	Mm00441941_m1		
	Fth	ferritin heavy chain	Mm00850707_g1		
	Ftl	ferritin light chain	Mm03030144_g1		
	Gapdh	glyceraldehyde-3-phosphate dehydrogenase	Mm99999915_g1		

### 2.10 Cultured photoreceptor cells and iron chelator

661W cells, a mouse photoreceptor cell line, were kindly donated by Dr. Muayyad R. Al-Ubaidi (University of Oklahoma Health Sciences Center, Oklahoma City, OK, USA). Cells were maintained in Dulbecco's modified Eagle's medium (Sigma-Aldrich, St. Louis, MO, USA) containing 10% fetal bovine serum (Gibco, Waltham, MA, USA) and 1% penicillin–streptomycin (Merck KGaA, Darmstadt, Germany). To determine the effect of iron chelators against *T. gondii* infection in Vero cells and 661W cells, 5 × 10^5^ parasites/mL of luciferase-expressing *T. gondii* (RH-Luc) [37] were incubated for 4 h, followed by incubation with DFP, Atovaquone (Sigma). After 48 h, 100 μL of Steady-Glo Luciferase Assay System (Promega) was added and bioluminescence was measured (SpectraMax iD5 Multi-Mode Microplate Readers, Molecular Device).

Parasite growth was calculated using the following formula:

$$\%\;Growth=\frac{\left(luminescence\;level\;of\;sample-background\right)}{\left(average\;of\;luminescence\;level\;of\;control-average\;background\right)}\times 100\%$$

### 2.11 Statistical analysis

We expressed the data as mean ± standard deviation (SD; n = number of samples). In cases where one patient received treatment for both their right and left eyes, we counted each eye individually (n = 2). For human and mouse samples, we used the Mann–Whitney *U* test to compare the two groups. To compare more than two groups, we used the Dunnett's tests. To perform *in vitro* assay using cultured cells, two-way ANOVA was conducted. *P*-values of <0.05 were considered statistically significant in all analyses. The Statistical Package for the Social Sciences software version 29 (IBM Inc.) was used.

## 3 Results

### 3.1 Intravitreal iron decrease and iron detection in the retina of patients with OT

**Fig. 1:**
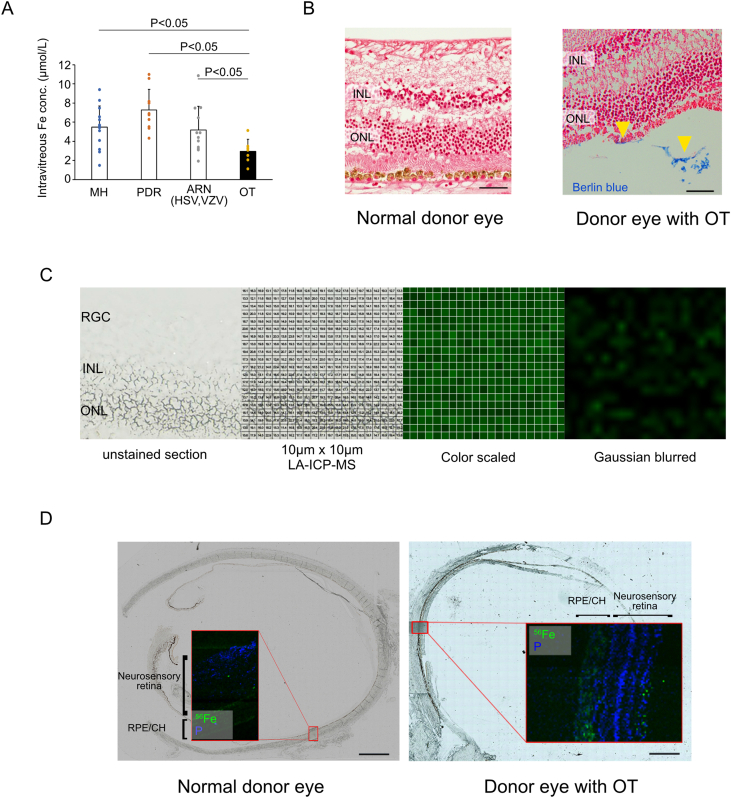
Detection of intravitreal and intraretinal iron from human patients with OT (A) Iron levels in the VH from eyes with OT (n = 7) were significantly lower than those from the MH (n = 13), PDR (n = 10), and ARN caused by HSV&VZV (n = 12). (B) Retinal sections obtained by autopsy from 25 y.o. Caucasian male with toxoplasmosis secondary to AIDS showing iron as confirmed by Berlin blue staining (yellow arrow heads), whereas normal donor eyes did not show Berlin blue staining positivity. (C) Representative visualization of iron (green) percentage compared to carbon in 10 μm × 10 μm square measured by LA–ICP–MS in retinal sections, through color scaled and Gaussian blurred. (D) Iron presence was confirmed by LA–ICP–MS in a donor eye from a patient with OT. Data are presented as mean ± SD. VH: vitreous humor, OT: ocular toxoplasmosis, MH: macular hole, PDR: proliferative diabetic retinopathy, ARN: acute retinal necrosis, HSV: herpes simplex virus, VZV: varicella zoster virus, INL: inner nuclear layer, ONL: outer nuclear layer, RPE/CH: retinal pigment epithelium/choroid. Scale bars = 50 μm (B) and 2 mm (D). (For interpretation of the references to color in this figure legend, the reader is referred to the Web version of this article.)

We compared vitreous iron concentrations in cases of macular hole (MH), proliferative diabetic retinopathy (PDR), acute retinal necrosis (ARN) caused by HSV/VZV, and OT. The iron level in eyes with OT was 2.96 ± 1.25 μmol/L (n = 7), which was significantly lower than that in eyes with MH 5.49 ± 2.21 μmol/L (n = 13, P = 0.021), PDR 7.27 ± 2.17 mmol/L (n = 10, P < 0.001), and ARN 5.18 ± 2.49 mmol/L (n = 12, P = 0.044) (Fig. 1A). Next, we attempted to detect iron in the retinal sections of cases infected with OT. Retinal sections obtained by an autopsy from a 25-year-old Caucasian male with OT secondary to AIDS showed the existence of iron, as confirmed by Berlin blue staining (Fig. 1B). In contrast, no clear locations of Berlin blue stain positivity were observed in the normal donor eye (Fig. 1B). Fig. 1C shows a representative illustration of the numerical values obtained by LA–ICP–MS converted specific color intensities, followed by being Gaussian blurred. Iron presence in the neurosensory retina was confirmed via LA–ICP–MS in a donor eye from a patient with OT (Fig. 1D). In contrast, no apparent ^56^Fe-positive sites in the neurosensory retina were evident in the normal donor eye (Fig. 1D). Phosphorus, an important marker for location identification, is ubiquitously present in all tissues [38,39]. Its distribution in the eye is quite similar to that in the nucleus of the retina [40].

### 3.2 Iron uptake into the outer retinal layer after *T. gondii* infection

**Fig. 2:**
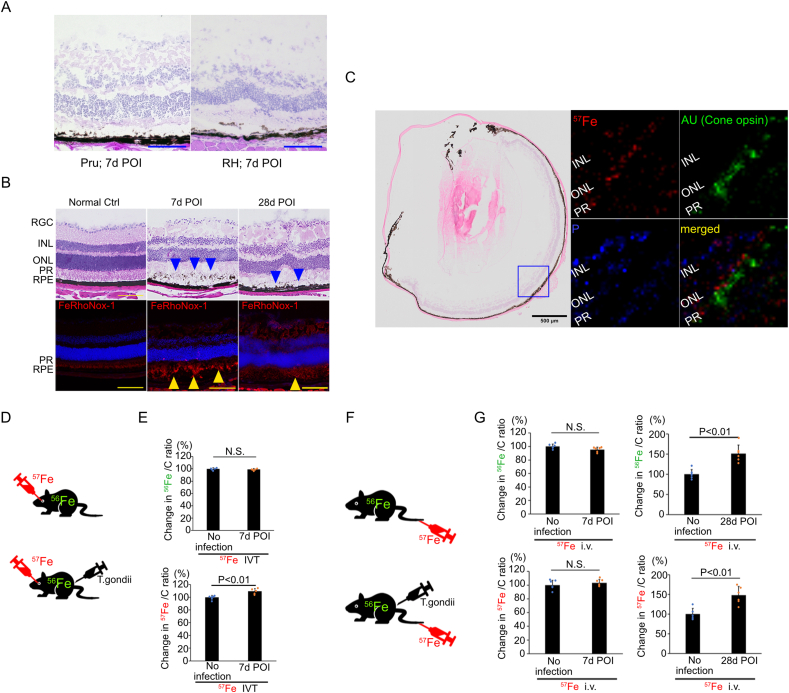
Iron uptake into *T. gondii*-infected mouse eyes (A) Comparison of Pru and RH strain of *T. gondii*-infected mouse eyes showed similar toxoplasma retinochoroiditis at 7days POI. (B) *T. gondii*-infected mouse eyes showed toxoplasmic retinochoroiditis (blue arrow heads) and Fe(II) accumulation as detected by FeRhoNox-1 (red) in PRL at 7 and 28 days POI (yellow arrow heads). (C) LA–ICP–MS detected a colloidal gold-positive (Cone opsin-positive) site and revealed the localization of ^57^Fe in the retina of ^57^Fe IVT mice. Note that ^57^Fe mostly accumulated at locations adjacent to the inner/outer segment of photoreceptor cells. (D) ^57^Fe was administered intravitreally (IVT) simultaneous to *T. gondii* infection.(E) The ^56^Fe/C ratio in the retina of *T. gondii*-infected mice was not significantly different from that in noninfected eyes, whereas the ^57^Fe/C ratio in the retina of *T. gondii*-infected mouse was significantly higher than that in noninfected eyes at 7 days POI (n = 6). (F) ^57^Fe was administered intravenously (i.v.) simultaneous to *T. gondii* infection. (G) The ^56^Fe/C ratio in the retina of *T. gondii*-infected mice was not significantly different from that in noninfected eyes, and the ^57^Fe/C ratio in the retina of *T. gondii*-infected mice was not significantly different from that in noninfected eyes at 7 days POI. Both ^56^Fe/C and ^57^Fe/C ratio in the retina of *T. gondii*-infected mice was significantly higher than that in noninfected eyes at 28 days POI. (n = 6) Data are presented as mean ± SD. AU: gold, INL: inner nuclear layer, ONL: outer nuclear layer, PR: photoreceptor, RGC: retinal ganglion cell, RPE: retinal pigment epithelium, POI: post infection, N.S.: there was no significant difference, IVT: intravitreal injection, i.v.: intravenous injection, Scale bar = 100 μm in (A). (For interpretation of the references to color in this figure legend, the reader is referred to the Web version of this article.)

For the detailed analysis of the changes observed in human eyes with OT, we employed the mouse OT model. We observed that both *T. gondii* (Pru)-infected and *T. gondii* (RH)-infected mice developed toxoplasmic retinochoroiditis at 7days POI (Fig. 2A); thus, we used *T. gondii* (Pru) for this study. FeRhoNox-1-positive areas (red), which indicate catalytic Fe^2+^ accumulation, were identified in the outer retina, consistent with the areas of inflammation observed with hematoxylin and eosin staining in *T. gondii*-infected mouse eyes at 7 and 28 days POI (Fig. 2B). Because only a small amount of ^57^Fe exists in nature [27], it is assumed that ^57^Fe detected *via* LA–ICP–MS, was the one administered for experimental purposes. Simultaneous administration of ^57^Fe and induction of *T. gondii* infection is considered to accurately depict the iron dynamics in mice. Thus, we performed ^57^Fe IVT in the mouse eyes simultaneously to *T. gondii* infection. Thereafter, LA–ICP–MS detected ^57^Fe in the retina of *T. gondii*-infected mice. To detect ^57^Fe localization in the retina of ^57^Fe IVT mice eyes, we labeled cone opsin with a colloidal gold-conjugated antibody. LA–ICP–MS revealed that ^57^Fe was mostly accumulated at locations adjacent to the opsin-positive site (mainly the inner/outer segment of photoreceptor cells) by detecting the colloidal gold (Fig. 2C). While the ^56^Fe/C ratio in the retina of *T. gondii*-infected mice (99.2% ± 1.5%, n = 6) was not significantly different from that in noninfected eyes (100.0% ± 2.0%, n = 6, P = 0.59) at 7 days POI, the ^57^Fe/C ratio in the retina of *T. gondii*-infected mice (109.3% ± 4.3%, n = 6) was significantly higher than that in noninfected eyes (100.0% ± 3.1%, n = 6, P = 0.004) (Fig. 2D and E). These results indicate that intravitreal iron reached the neurosensory retina after infection. Similarly, we administered ^57^Fe i.v. simultaneously to *T. gondii* infection. The ^56^Fe/C ratio in the retina of *T. gondii*-infected mice (95.4% ± 3.6%, n = 6) was not significantly different from that in noninfected eyes (100.0% ± 3.6%, n = 6, P = 0.055), nor was the ^57^Fe/C ratio in the retina of *T. gondii*-infected mice (103.2% ± 45.0%, n = 6) from that in noninfected eyes (100.0% ± 7.0%, n = 6, P = 0.337) at 7 days POI. However, the ^56^Fe/C ratio in the retina of *T. gondii*-infected mice (151.1% ± 21.5%, n = 6) was significantly higher than that in noninfected eyes (100.0% ± 11.8%, n = 6, P = 0.004), as was the ^57^Fe/C ratio in the retina of *T. gondii*-infected mice (148.4% ± 22.0%, n = 6) compared to that in noninfected eyes (100.0% ± 14.4%, n = 6, P = 0.006) at 28 days POI (Fig. 2F and G).

### 3.3 Retinal ferroptosis in *T. gondii*-infected mouse eyes

**Fig. 3:**
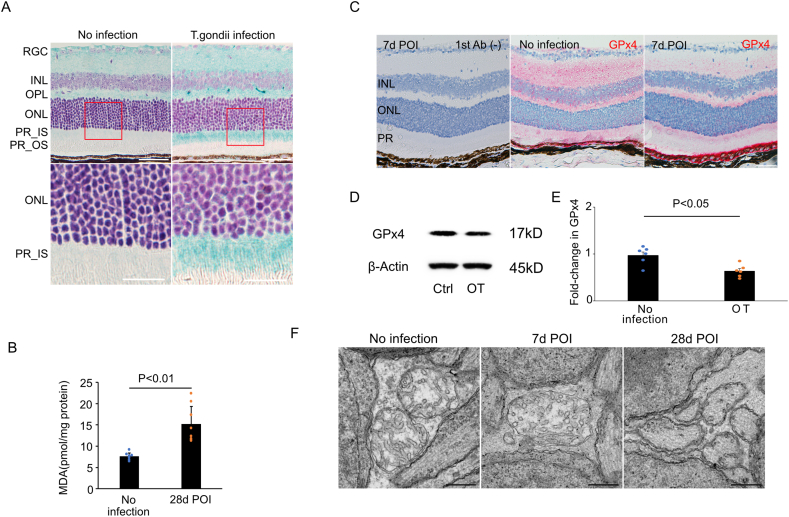
Retinal ferroptosis in *T. gondii*-infected mouse eyes (A) Immunohistochemistry of mouse retina in *T. gondii*-infected and noninfected eyes for detection of oxidative stress using 4-HNE antibodies. Cell nuclei are shown in blue. Note that 4-HNE-positive cells (immunostained with HistoGreen) are the most prominent in the PR_IS of the *T. gondii*-infected eye. (B) The MDA levels in *T. gondii*-infected (n = 8) mouse retinas were significantly higher than those in noninfected retinas (n = 8) (C) Representative GPx4 immunostaining on *T. gondii*-infected mouse retinal section. The GPx4 expression in *T. gondii*-infected retina was decreased compared to that in noninfected retinas at 7 days POI. (D) Western blots show lower GPx4 expression in OT than in noninfected eyes at 7 days POI (E) Quantitative densitometry results of the Western blotting data of (D) normalized to the intensity of β-actin (n = 6) (F) TEM showing smaller mitochondria in photoreceptor cells from eyes with *T. gondii* infection and loss of cristae compared to noninfected mice at 7 and 28 days POI. Data are presented as mean ± SD. INL: inner nuclear layer, ONL: outer nuclear layer, PR: photoreceptor, RGC: retinal ganglion cell, RPE: retinal pigment epithelium, IS: inner segment, OS: outer segment, 4-HNE: 4-hydroxy-2-nonenal, MDA: malondialdehyde, GPx4: glutathione peroxidase 4, POI: post infection, N.S.: there was no significant difference, TEM: transmission electron microscopy, OT: ocular toxoplasmosis. Scale bar = 50 μm in (A). in (C) in (E). (For interpretation of the references to color in this figure legend, the reader is referred to the Web version of this article.)

Based on the above, we hypothesized that the iron uptake observed in *T. gondii* infection is associated with ferroptosis. Fenton reaction-based oxidative damage, which can be detected using 4-HNE, is strongly involved in ferroptosis [41]. Retinal sections from the eyes of mice infected with *T. gondii* exhibited prominent positive staining of 4-HNE, especially in the inner segment of photoreceptor (Fig. 3A). The level of another oxidative stress marker, MDA, was significantly higher in the retina of *T. gondii*-infected mouse eyes than in the noninfected eyes (15.18 ± 4.12 pmol/mg [n = 8] vs. 7.61 ± 0.78 pmol/mg protein [n = 8], p < 0.001) at 28 days POI (Fig. 3B). GPx4 is a multifunctional protein which can reduce peroxidized lipids either in free form or in complex with lipids, having a predominant role in preventing ferroptosis [42]. Immunohistochemistry analysis showed lower GPx4 expression in the retina of *T. gondii*-infected mice than in that of noninfected mice at 7 days POI (Fig. 3C). Melanin-rich aggregation was observed in the RPE monolayer of *T. gondii*-infected mice. Western blot analysis of the retina also revealed decreased GPx4 expression in the *T. gondii*-infected mice (63.5% ± 12.0%, n = 6) in comparison to the noninfected mice (97.0% ± 17.2%, n = 6, P = 0.01) at 7 days POI (Fig. 3D and E). Transmission electron microscopy (TEM) showed that the mitochondria in photoreceptor cells from eyes with *T. gondii* infection displayed a significantly smaller size and a significant cristae loss compared to the control noninfected mice (Fig. 3F), which are consistent with the changes in the mitochondria of cells undergoing ferroptosis.

### 3.4 Iron-related gene and protein analyses

**Fig. 4:**
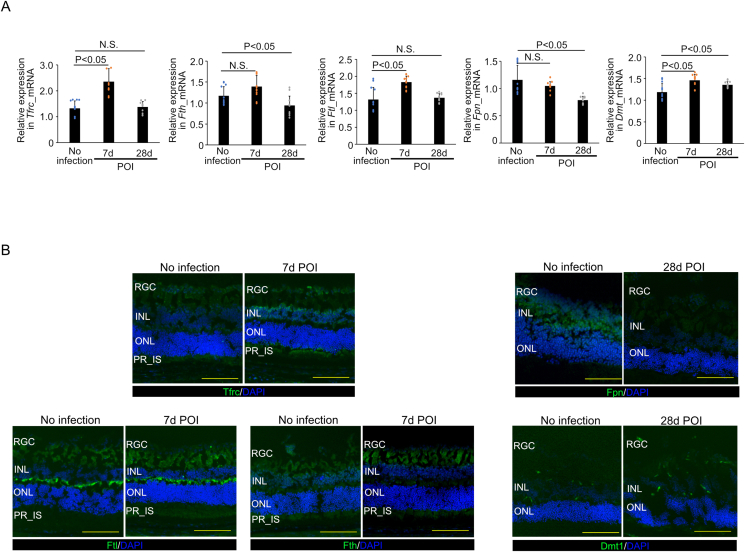
Iron-related gene and protein analyses (A) *Tfrc*, *Ftl*, and *Dmt1* mRNA expression in *T. gondii*-infected retinas were significantly higher than those in noninfected mice at 7 days POI (n = 12). The *Fth* and *Dmt1* mRNA expressions at 28 days POI in mice with *T. gondii*-infected retinas were significantly lower and higher than those in mice without infection (n = 12), respectively. Furthermore, the *Fpn* mRNA expression at 28 days POI in mice with *T. gondii*-infected retinas was significantly lower than that of mice without infection (n = 12). (B) Immunohistochemistry showing increased Tfrc positivity and unchanged Ftl, Fth levels at 7days POI, and reduced Fpn positivity and unchanged Dmt1 levels at 28 days POI. Data are presented as mean ± SD. INL: inner nuclear layer, ONL: outer nuclear layer, PR: photoreceptor, RGC: retinal ganglion cell, IS: inner segment, POI: post infection, N.S.: no significant difference, Scale bars = 100 μm in (B).

Next, we assessed the cellular iron transport and export pathways. The relative expression of *Tfrc* mRNA in the retina was 1.33 ± 0.28, 2.36 ± 0.50, and 1.38 ± 0.21 in noninfected mice, *T. gondii*-infected mice at 7 days POI, and *T. gondii*-infected mice at 28 days POI, respectively (n = 12/group). *Tfrc* mRNA expression in *T. gondii*-infected mice at 7 days POI was significantly higher than that in noninfected mice (P < 0.001), but that at 28 days POI did not show significant difference (Fig. 4A). The relative expression of *Fth* mRNA in the retina was 1.17 ± 0.21, 1.39 ± 0.26, and 0.94 ± 0.22 in the noninfected mice, *T. gondii*-infected mice at 7 days POI, and *T. gondii*-infected mice at 28 days POI, respectively (n = 12/group). *Fth* mRNA expression in *T. gondii*-infected mice at 28 days POI was significantly lower than that in noninfected mice (P = 0.042, Fig. 4A). The relative expression of *Ftl* mRNA was 1.32 ± 0.35, 1.83 ± 0.18, and 1.37 ± 0.12 in the noninfected mice, *T. gondii*-infected mice at 7 days POI, and *T. gondii*-infected mice at 28 days POI, respectively (n = 12/group). *Ftl* mRNA expression in *T. gondii*-infected mice at 7 days POI was significantly higher than that in noninfected mice (P < 0.001, Fig. 4A). The relative expression of *Fpn* mRNA was 1.16 ± 0.27, 1.05 ± 0.08, and 0.79 ± 0.07 in noninfected mice, *T. gondii*-infected mice at 7 days POI, and *T. gondii*-infected mice at 28 days POI, respectively (n = 12/group). *Fpn* mRNA expression in *T. gondii*-infected mice was gradually decreased after infection and that at 28 days POI was significantly lower than that in noninfected mice (P < 0.001, Fig. 4A). The relative expression of *Dmt1* mRNA was 1.19 ± 0.19, 1.46 ± 0.14, and 1.35 ± 0.07 in noninfected mice, *T. gondii*-infected mice at 7 days POI, and *T. gondii*-infected mice at 28 days POI, respectively (n = 12/group). *Dmt1* mRNA expression at 7 and 28 days POI in mice with *T. gondii* infection was significantly higher than that in the mice without infection (P < 0.001 and P = 0.15, respectively) (Fig. 4A). Next, we evaluated changes in Tfrc, Ftl, Fth, Fpn, and Dmt1 protein expression in the retina after *T. gondii* infection via immunohistochemistry. This experiment showed increased Tfrc expression but no changes in Ftl and Fth in infected retinas at 7 days POI and decreased Fpn expression and unchanged Dmt1 levels at 28days POI (Fig. 4B). Collectively, the analysis of mRNA and protein expression revealed upregulation of Tfrc and downregulation of Fpn.

### 3.5 Deferiprone protects against toxoplasmic retinochoroiditis and cultured photoreceptor cells

**Fig. 5:**
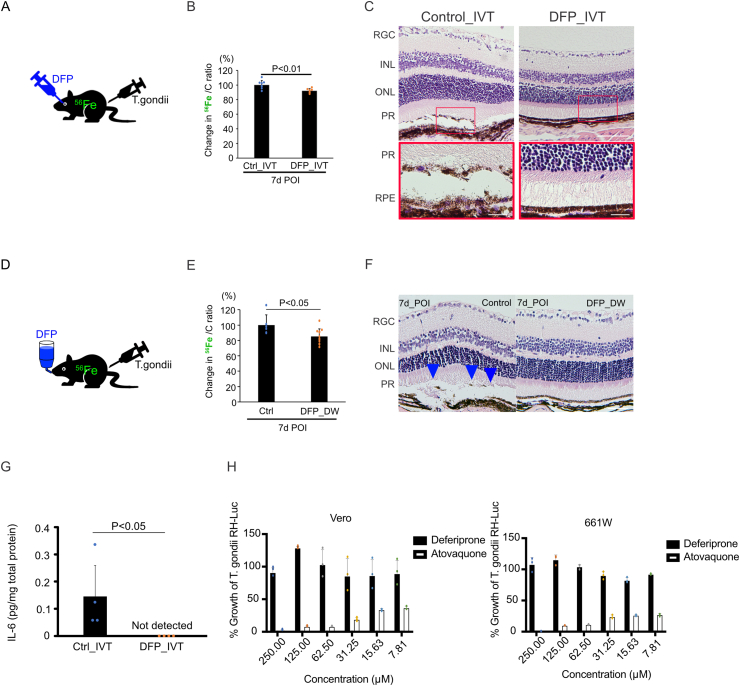
Iron chelator ameliorated toxoplasmic retinochoroiditis (A) DFP was administered intravitreally (IVT) simultaneous to *T. gondii* infection. (B) Compared to the control PBS-injected eye, DFP-injected (IVT) eyes showed significantly reduced ^56^Fe accumulation at 7 days POI (n = 9). (C) The DFP-injected (IVT) eyes did not show retinochoroiditis at 7 days POI, whereas the control PBS-injected eyes showed retinochoroiditis (enlarged images). (D) DFP was administered per os in drinking water 3–7 days before *T. gondii* infection. (E) DFP-drinking water (DW) mice showed significantly lower ^56^Fe accumulation at 7 days POI (n = 9). (F) The DFP-DW mice did not show retinochoroiditis at 7 days POI, whereas the control mice had retinochoroiditis (blue arrow heads). (G) Intraretinal IL-6 levels were significantly reduced in DFP-injected (IVT) eyes at 7 days POI (n = 4). (H) DFP treatment for *T. gondii*-infected Vero and 661W cells did not result in significant suppression in *T. gondii* growth compared to other drug for OT. Data are presented as mean ± SD. DFP: deferiprone, POI: post infection, INL: inner nuclear layer, ONL: outer nuclear layer, PR: photoreceptor, RGC: retinal ganglion cell, RPE: retinal pigment epithelium, IVT: intravitreal injection, DW: drinking water. Scale bar: 100 μm (upper) & 20 μm (lower) in (C), and 100 μm in (F). (For interpretation of the references to color in this figure legend, the reader is referred to the Web version of this article.)

We investigated the potential of DFP treatment for OT to prevent toxoplasmic retinochoroiditis. Compared to PBS-injected (IVT) eyes (100.0% ± 6.2%, n = 9), DFP-injected (IVT) eyes showed significantly reduced ^56^Fe accumulation (92.0% ± 3.0%, n = 9, P = 0.005) at 7 days POI (Fig. 5A and B). Interestingly, DFP-injected (DFP_IVT) eyes did not show retinochoroiditis and the RPE monolayer was well organized at 7 days POI (Fig. 5C). In addition, we mixed DFP with drinking water to examine the effect of oral medication. DFP in drinking water (DFP_DW) mice showed significantly lower ^56^Fe accumulation (85.2% ± 9.8%, n = 12, P = 0.03) at 7 days POI than control mice (100.0% ± 13.5%, n = 6) (Fig. 5D and E). Moreover, the eyes of DFP_DW mice did not show retinochoroiditis at 7 days POI (Fig. 5F). We also investigated IL-6 in the neurosensory retina of *T. gondii*-infected eyes with or without DFP_IVT. Compared with the IL-6 level in PBS-injected eyes (0.15 ± 0.11 pg/mg total protein, n = 4), the level in DFP-injected eyes was significantly lower (not detected, n = 4. P = 0.01) (Fig. 5G). The percentage of growth of *T. gondii* RH-Luc in Vero cells (mean ± SD [drug concentration]) incubated with DFP (n = 3) at 48 h was 89.3 ± 20.5 [7.81 μM], 86.3 ± 24.2 [15.63 μM], 85.6 ± 26.8 [31.25 μM], 103.1 ± 23.1 [62.5 μM], 128.7 ± 2.1 [125 μM], and 91.0 ± 7.3 [250 μM], whereas that in Atovaquone (n = 3) was 36.8 ± 2.7 [7.81 μM], 33.9 ± 1.9 [15.63 μM], 26.8 ± 3.0 [31.25 μM], 23.1 ± 0.5 [62.5 μM], 2.1 ± 0.9 [125 μM], and 2.5 ± 0.9 [250 μM]. Similarly, the percentage of growth of *T. gondii* RH-Luc in 661W cells incubated with DFP (n = 3) at 48 h was 92.1 ± 0.9 [7.81 μM], 82.4 ± 4.4 [15.63 μM], 90.0 ± 6.3 [31.25 μM], 104.0 ± 3.9 [62.5 μM], 115.3 ± 7.9 [125 μM], and 107.8 ± 11.2 [250 μM]. In contrast the percentage of growth in Atovaquone (n = 3) was 27.2 ± 2.3 [7.81 μM], 26.2 ± 1.3 [15.63 μM], 23.9 ± 2.9 [31.25 μM], 11.1 ± 0.7 [62.5 μM], 9.9 ± 0.2 [125 μM], and 0.5 ± 0.2 [250 μM] (Fig. 5H). Atovaquone showed dose-dependent inhibitions of *T. gondii* RH-Luc growth in both Vero and 661W cells, whereas DFP showed no growth inhibition. These results indicated that DFP ameliorated toxoplasmic retinochoroiditis by reducing intraretinal iron accumulation and retinal inflammation but not by preventing *T. gondii* growth in the retina.

## 4 Discussion

The eye is one of the few organs from which clear aqueous humor (AH) can be safely collected, as well as serum/plasma, due to its excellent transparency. However, changes in iron concentration in the vitreous during infection have not been studied. To the best of our knowledge, only three published reports have assessed vitreous iron concentrations of the human eyes. These studies analyzed medical–legal autopsy samples [43] and examined the biochemical composition of living patients [44,45]. Compared with their respective average iron concentrations, OT samples in our study may have a lower iron concentration. However, this finding is not statistically significant. Conversely, vitreous iron levels in OT were significantly lower than those in retinal diseases such as macular hole (MH), a noninfectious, noninflammatory disease, and proliferative diabetic retinopathy (PDR), a noninfectious inflammation-associated disease characterized by elevated vitreous levels of key inflammatory cytokine markers such as IL6 and MCP-1 [46,47]. Of note, the vitreous iron level was not decreased in acute retinal necrosis (ARN), a representative virus-related disease. The difference in iron levels between ARN and OT may be key for elucidating new pathogenesis and proposing new diagnosis methods. To the best of our knowledge, this is the first report that detected a lower iron concentration in the VH of patients with OT. We confirmed iron uptake in the retina during *T. gondii* infection using LA–ICP–MS. A previous study subjected neurosensory retina from seven normal donor eyes to applied LA–ICP–MS and detected iron in only one sample [40]. Hence, the presence of iron in the neurosensory retina that we detected in the eye with OT is not commonly observed. The retina has a significantly elevated oxygen consumption due to its metabolic demands. Retinal photoreceptor cells are fatty acid-rich and highly oxidatively stressed tissues, as photoreceptor cells exposed to daylight accumulate lipids over time [48,49]. In addition, photoreceptors exhibit a high energy metabolism and associated oxygen consumption [50]. The daily photoreceptor disk recycling process, required by oxidative stress-induced harm, is facilitated by RPE cells via phagocytosis [51,52]. Prolonged disruption of this recycling mechanism can lead to dysfunction of both RPE and photoreceptors, resulting in the formation of lipid peroxidation byproducts, particularly 4-HNE and MDA [53]. Thus, retinal photoreceptor cells are extremely likely subjected to lipid accumulation and oxidation, which are the two major factors in ferroptosis. The involvement of ferroptosis in OT was confirmed by high levels of the lipid peroxidation products 4-HNE and MDA in *T. gondii*-infected mouse retinas. As GPx4 plays a crucial role in protection against ferroptosis [23,42], we measured its expression via immunohistochemistry and Western blotting. Western blot analysis showed lower GPx4 expression in *T. gondii*-infected mice retinas; similarly, immunohistochemistry showed particularly decreased GPx4 expression in *T. gondii*-infected retina in photoreceptor outer segment (PR_OS). Conversely, 4-HNE-positive cells were the most prominent in the photoreceptor inner segment (PR_IS) of the *T. gondii-*infected eye. The RPE monolayer exhibited morphological changes in *T. gondii*-infected eye. However, there were no differences in 4-HNE or GPx4 expression. Retinal ferroptosis might affect the neurosensory retina. Thus, we observed a reduction in the size and a loss of cristae in mitochondria in photoreceptor cells compared with the noninfected mice at 7 and 28 days POI, which are consistent with the changes occurring in the mitochondria of cells undergoing ferroptosis [54]. In addition, RT-PCR revealed elevated *Tfrc* and *Ftl* levels at 7 days POI. However, immunostaining showed a significant increase only in Tfrc. Meanwhile, alterations in Ftl and Fth levels were not evident. Furthermore, RT-PCR revealed that the *Fpn* expression significantly decreased at 28 days POI, and the corresponding immunostaining revealed a general attenuation in the widespread expression of Fpn across the neurosensory retina. Conversely, the *Dmt1* levels were elevated at both 7 and 28 days based on the RT-PCR results. Previous reports have not definitively elucidated the role of Dmt1 in the retina [52,55]. Transferrin and transferrin receptors are the principal pathways via which cells uptake iron [56]. Ferritin, comprising both light chain (Ftl) and heavy chain (Fth), plays an important role in representing the intracellular iron storage capacity, and excess iron is stored within ferritin [57,58]. As required, iron is transported out of cells via ferroportin (Fpn), the only known iron exporter [59]. Dmt1, located on the endosome, facilitates the transport of Fe^2+^ from the endosome into the cytoplasm [52,60]. Therefore, these results imply the promotion of iron intracellular storage in the retina.

Previous studies suggested that the iron chelators had a positive effect in *T. gondii* infection [13]. Furthermore, iron chelators exerted protective effects against ferroptosis [61]. DFP, deferoxamine, and deferasirox are the most commonly prevailing important US FDA-approved iron chelators. Deferoxamine reportedly induces retinal degeneration [62], whereas DFP was confirmed to have low toxicity in humans and mice [63,64]. Therefore, we used DFP in this study. We intravitreally injected DFP or administered it per os in drinking water. We confirmed that both DFP_IVT and DFP_DW reduced ^56^Fe accumulation and prevented retinochoroiditis [65]. IL-6 measurements in the retina of infected mice and *in vitro* experiments indicated that the therapeutic effect of DFP against OT is due to reduced inflammation but not due to decreased *T. gondii* growth.

The limitations of this study are as follows: (1) Although we have successfully detected ferroptosis across the whole neurosensory retina, the specific cell type engaged in this process remains unidentified yet. (2) Although intravitreal iron was taken up in the *T. gondii*-infected retina, it has not been elucidated whether the retinal cells take up iron or whether *T. gondii* that invades the retina take up iron. (3) In the present study, type II *T. gondii* was used to induce OT, and DFP showed a prophylactic effect on toxoplasmic retinochoroiditis induced by type II *T. gondii*, but whether the therapeutic effect of DFP is maintained for OT caused by type I *T. gondii* remains unclear. (4) The human retinal sections are old and difficult to acquire, which complicates the attainment of statistically significant differences. Furthermore, the resolution of LA–ICP–MS is challenging to enhance as laser ablation occurs at 10-μm intervals. (5) The invasion mechanism of *T. gondii* into the retina remains unclear, and the timing of *T. gondii* infection of the retina is not always the same as the timing of treatment. Human vitreous samples are less consistent with the timing of infection, as opposed to mice, in which samples are collected at a scheduled timing. In most cases, OT is acquired via infection [66], while it has been reported that many patients with choroidal scarring are unaware of the presence of the disease [67]. While it is interesting that the average iron concentration in vitreous humor was low in this context, we cannot rule out the possibility that similar results may not be obtained depending on the timing of diagnosis. Further research is required to elucidate these issues to apply iron-targeted new diagnostic and therapeutic methods to clinical practice.

## 5 Conclusion

In conclusion, our study demonstrates decreased iron levels in the vitreous humor (VH) of patients with OT and intraretinal accumulation of iron in the *T. gondii*-infected mouse eyes. LA–ICP–MS revealed intravitreal iron was taken up in the retina following infection. Biological examinations demonstrated increased oxidative stress, decreased GPx4, and mitochondrial changes in the *T. gondii*-infected mouse retina, indicating the involvement of ferroptosis. Furthermore, DFP successfully ameliorated toxoplasmic retinochoroiditis. Understanding the pathological role of ferroptosis in OT may potentially advance future diagnostic methods and treatment strategies.

## Declaration of generative AI in scientific writing

AI was not used for writing.

## Funding

This work was partially supported by Grants-in-Aid for Scientific Research C [H.K.; 22K09810]; and for young scientist [A.S.; 22K16949, and H.S.; 22K16968] from the 10.13039/501100001700Ministry of Education, Culture, Sports, Science and Technology (KAKENHI) (http://www.jsps.go.jp/); 10.13039/100007449Takeda Science Foundation (H.K.); the 10.13039/100007428Naito Foundation (A.S.); and the joint research grants from the National Research Center for Protozoan Diseases, Obihiro University of Agriculture and Veterinary Medicine (2022-joint-1, 2023-joint-2). This work was supported in part by 10.13039/501100003382JST CREST (JPMJCR19H4) and 10.13039/501100001691JSPS Kakenhi (JP19H05462 and JP20H0550) to ST.

## Declaration of competing interest

The authors declare that they have no known competing financial interests or personal relationships that could have appeared to influence the work reported in this paper.
